# A Spectroscopic Study of Tautomeric Equilibrium of Salicylideneaniline in ZSM-5 Zeolites

**DOI:** 10.3390/molecules24040795

**Published:** 2019-02-22

**Authors:** Matthieu Hureau, Alain Moissette, Konstantin S. Smirnov

**Affiliations:** LASIR—Laboratoire de Spectrochimie Infrarouge et Raman, CNRS, UMR 8516, University of Lille, F-59000 Lille, France; Matthieu.Hureau@univ-lille.fr

**Keywords:** salicylideneaniline, zeolite, ZSM-5, tautomer, equilibrium, spectroscopy, DFT calculations

## Abstract

Salicylideneaniline (SA) sorbed in cation-exchanged M-ZSM-5 (M = H^+^, Li^+^, Na^+^, K^+^, Rb^+^, Cs^+^ and Zn^2+^) zeolites was studied by spectroscopic techniques assisted by quantum-chemical calculations. The nature of extra-framework cations present in the zeolite void was found to affect the spectral signature of the sorbed SA molecule that points to the shift of tautomeric equilibrium between the enol and keto forms. Small size cations, such as H^+^ and Li^+^, stabilize a cis-keto SA tautomer along with a enol one in the zeolite structure. The calculations indicate that the sorbed cis-keto tautomer may have the dipole large enough to be considered as a zwitterion. New features appearing in the spectra with the increase of the cation size were attributed to the presence of trans-keto SA tautomer, which up to now has been observed only in time-resolved spectroscopic experiments. A strong interaction of the molecule with cations in Zn-ZSM-5 zeolite results in the chelation of enol SA with the divalent Zn^2+^ ions. The results of the study suggest that the tautomeric equilibrium of molecules belonging to the Schiff base family can be tuned by the confinement in the nanoporous materials via a choice of topology of zeolite framework and the nature of extra-framework cations.

## 1. Introduction

Among molecules belonging to the Schiff base family, N-salicylideneaniline (SA) is a model compound largely studied in both the solid state [1,2,3,4,5] and solutions with various solvents [3,5,6,7,8,9,10,11,12,13]. Due to its photochromic, thermochromic and solvatochromic properties, this molecule is of significant interest for potential applications in various domains from optical devices [14,15,16] and molecular switches [17,18,19] to medicine [20,21,22]. SA is well known to adopt an enol form in the ground state. Photo- and thermal excitation of the molecule lead to an excited state intramolecular proton transfer producing an excited cis-keto form, (cis-keto)${}^{*}$, with a possible zwitterionic structure [3,9,10,13,23,24,25]. This form then returns to the ground state enol SA conformer via either (cis-keto)${}^{*}\to \;$ cis-keto deactivation or (cis-keto)${}^{*}\to \;$ trans-keto isomerization followed by a conversion to the cis-keto tautomer in the ground state [5,25,26,27,28]. Results of quantum-chemical calculations generally confirm this scenario [25,29,30].

The molecule’s environment can significantly influence the equilibrium between the conformers that can lead to the co-existence of several forms [5,31,32,33]. Consequently, several attempts have been undertaken to shift the equilibrium towards a particular tautomer. For instance, Bogdan et al. [34] obtained the displacement of the equilibrium between the phenolic tautomer and the quinoid one towards the keto form by adding ethanol into cyclohexane/ethanol mixture that increased the solvent’s polarity. To control photo-activity at the molecular level and to tune the effects of environment, salicylideneaniline and its derivatives were incorporated into specific nanoporous and mesoporous hosts, such as zeolites (typically faujasite) [23,24,35,36,37], mesoporous molecular silica (MCM-41 and SBA) [12,38,39,40], polymer matrix [41] and into micelles [12]. All these host structures provide an environment with a different degree of confinement and polarity and the life-time of transient species created upon photo-excitation after the encapsulation was found to depend on the pore size and the chemical composition of host. Thus, the cis-keto form of SA and of its derivatives was stabilized in MCM-41 mesoporous silica [38] and in Na-exchanged faujasite zeolites [23,24,35]. However, the host–guest interactions in these materials may lead to the co-existence of different conformers already in the ground state that complicates the investigation of the systems upon photo-excitation. To overcome this problem, the photodynamics of SA was studied after the incorporation of the molecule in photo-inert molecular capsules with weak host–guest interactions [27]. Another approach consisted in introducing SA into Al-free zeolites, where the molecule can exist in a quasi-isolated state because of an inert environment of pure silica structure [42].

While there exists a large body of information on stable structural forms of SA and its derivatives, description of unstable conformers remains rather controversial. Indeed, if such forms as cis-keto tautomer were stabilized and characterized upon the incorporation in nanohosts, some short-lived transient species were observed only after a photo-excitation or their existence was inferred from results of time-resolved spectroscopic experiments. Consequently, such species have never been reliably characterized, particularly by means of spectroscopic techniques. The identification of these unstable intermediates requires a specific environment that favors a durable formation of the species and inhibits backward reactions.

In this study, to stabilize otherwise short-living forms of the SA molecule, we took advantage of a high degree of confinement and of a polar environment, which were provided by cation-exchanged MFI-type zeolites (ZSM-5). Indeed, these materials are known to behave as veritable solid solvents with controlled acid/basic properties [23,43]. The porous structure of MFI zeolites consists of a two-dimensional network of intersecting channels: the straight channels with a cross-section of 0.53 nm × 0.56 nm and the zigzag ones with 0.51 nm × 0.55 nm openings. The size of the channels provides a tight fit of rod-shaped poly-aromatic molecules in the pores and a local polarity of the host lattice can be adjusted by varying the nature of exchangeable charge-balancing cations present in the zeolite void. The investigation of the tautomeric equilibrium of N-salicylideneaniline sorbed in M-ZSM-5 zeolites with charge-balancing cations M = H^+^, Li^+^, Na^+^, K^+^, Rb^+^, Cs^+^ and Zn^2+^ was carried out by complementary spectroscopic methods, such as diffuse reflectance UV-vis absorption, vibrational Raman scattering and infrared spectroscopies. Changes in the spectra as a function of the cation size and charge, and their comparison with the counterpart data obtained for SA sorbed in an Al-free ZSM-5 zeolite (silicalite-1) shed light on the equilibrium between SA tautomers and enabled us to attribute some of the observed spectral features to molecular species, which until now were never stabilized in either solutions or confined media. The interpretation of the spectral data was assisted by quantum-chemical calculations.

## 2. Materials and Methods

### 2.1. Materials

A purely siliceous MFI sample (silicalite-1) synthesized in a fluoride medium was a gift of Dr. Joël Patarin (Institut de Science des Matériaux, Mulhouse, France). Na-ZSM-5 and H-ZSM-5 zeolite samples (Si/Al = 13.5, average particle size ∼1 μm) were obtained from VAW aluminium (Schwandorf, Germany). Salicylideneaniline (SA) was obtained from Sigma Aldrich (97%) in a powder form and was used as is. To avoid the water contact with the hydrophilic ZSM-5 zeolites and with the SA molecule, the samples were manipulated in an argon atmosphere.

#### 2.1.1. Cation-Exchanged ZSM-5 Zeolites

To prepare M-ZSM-5 zeolites with M = Li^+^, K^+^, Rb^+^, Cs^+^, Zn^2+^, the sodium extra-framework cations of Na-ZSM-5 were exchanged by using the corresponding chloride salt (100 mL, 0.1 mol L${}^{-1}$). The exchange process was carried out by suspending zeolite powder in MCl (M = Li^+^, K^+^, Rb^+^, Cs^+^) and ZnCl_2_ aqueous solution under stirring. The solid phase was filtered off after 24 h and dried at 200 °C in an oven for 12 h, then stirred again with a fresh solution of the chloride salt and dried. The procedure was repeated four times. The resulting solid phase was washed by de-ionized water, isolated, dried at 200 °C for 12 h, and then calcined at 450 °C in air for 6 h. The elementary analyses of so-prepared zeolites indicated that the Na cations of the parent material were completely exchanged using the above procedure. Elemental analysis of the samples resulted in the following unit cell chemical composition: (SiO_2_)_96_ (silicalite-1), M_6.6_(AlO_2_)_6.6_(SiO_2_)_89.4_ (M = H^+^, Li^+^, Na^+^, K^+^, Rb^+^, Cs^+^), and Zn_3.3_(AlO_2_)_6.6_(SiO_2_)_89.4_. The chemical analysis, powder XRD patterns, ^29^Si and ^27^Al MAS-NMR, IR, Raman, and DRUVv spectra of the bare exchanged zeolites were characteristic of well-crystallized porous materials with a very low amount of extra-framework aluminium species.

#### 2.1.2. SA Loaded Zeolites

Weighed amounts (∼1.4 g) of the zeolite samples were introduced into an evacuable, heatable silica cell placed into a vertical oven connected to a vacuum network. The samples were stepwise heated up to 450 °C under a flow of dry Ar for 12 h. Then, they were cooled down to room temperature under dry argon. An amount of SA corresponding to one molecule per unit cell of M-ZSM-5 was introduced into the cell under dry argon atmosphere at room temperature and then the powder mixture was carefully shaken. After a homogeneous mixing, the powder was transferred under dry argon into a quartz glass Suprasil cell and sealed. All the samples were stocked in the sealed cells at 40 °C for six months in the dark. After the storing period, the UV-vis and Raman spectra of the samples did not show any evolution, thus indicating that the systems had reached equilibrium. In what follows, the SA@M-ZSM-5 notation stands for the SA loaded M-ZSM-5 zeolite.

After the mixing of SA with the M-ZSM-5 zeolites, a gradual change of powder color from yellowish to greenish (H^+^ and Li^+^) and to brownish (Na^+^, K^+^, Rb^+^, and Cs^+^) was observed. Contrarily, the SA@silicalite sample kept its initial yellowish color after the six-month period. The progressive color change of the SA@M-ZSM-5 powders suggests that the sorbed SA molecules adopted specific conformations after having diffused towards preferred sorption sites in the zeolite pore system. This observation is in line with results of confocal fluorescence imaging studies that have shown the penetration of organic dye molecules of size similar to that of SA to the depth of H-ZSM-5 zeolite single crystals [44,45,46].

### 2.2. Methods

#### 2.2.1. Diffuse Reflectance UV-Visible (DRUVv) Absorption Spectroscopy

The UV-visible absorption spectra of the samples were taken in the spectral range 200–1800 nm by using a Cary 6000 spectrometer. The instrument was equipped with an integrating sphere allowing to record the spectra of powdered samples stocked under argon in the quartz cells via diffuse reflectance; the corresponding dehydrated bare zeolite was used as the reference. The DRUVv spectra were plotted as the Kubelka–Munk function

(1) $$F\left(R\right)=\frac{{\left(1-R\right)}^{2}}{2R},$$

where *R* is the ratio of the diffuse reflectance of the SA-loaded zeolite to that of the reference material. F(R) was registered as a function of wavelength.

#### 2.2.2. Raman Spectrometry

Raman spectra were recorded using a near-IR FT-Raman spectrometer (Bruker RFS 100/S instrument) with a CW Nd:YAG laser ($\lambda_{0}=1064$ nm) as an excitation source. A laser power of 10–100 mW was used. The spectra were measured in the region 150–4000 cm${}^{-1}$ with a resolution of 2 cm${}^{-1}$ and were accumulated over 600 scans.

#### 2.2.3. Infrared Spectrometry

Diffuse reflectance infrared spectra (DRIFT) were recorded on a Thermo-Nicolet Magna 860 FTIR spectrometer equipped with a liquid nitrogen cooled MCT detector. The DRIFT spectra were registered with a resolution of 2 cm${}^{-1}$ and were plotted as the Kubelka–Munk function (Equation (1)) against wavenumber.

#### 2.2.4. Quantum-Chemical Calculations

**Figure 1:**
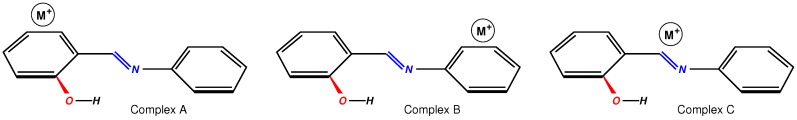
Initial configurations of salicylideneaniline–metal ion complexes.

Calculations of salicylideneaniline–metal ion complexes (denoted hereafter SA@M, M = Li^+^, Na^+^, K^+^, Zn^2+^) were performed at the DFT level with the use of the 6-311++G(d,p) basis set with the Gaussian 09 code [47]. Two hybrid exchange-correlation functionals were utilized in the calculations: B3LYP and ωB97X-D. The former is known to correctly describe the vibrational dynamics of both molecular and periodic systems and it was used in our previous study [42]. ωB97X-D XC is a long-range corrected functional with dispersion correction [48]. The functional gives an improved description of thermochemistry for system with covalent and non-covalent interactions. Three possible initial configurations were considered for the SA@M complexes. They are sketched in Figure 1 for the ground state enol SA conformer. Configurations A and B corresponded to the cation placed above the phenol and benzene ring of the molecule, respectively. In Configuration C (Figure 1), the cation was put above the C=N bond and positioned roughly perpendicular to the molecular plane. The choice of the initial configurations was dictated by the assumption that the cation would preferentially interact with the electron density of the phenol and benzene rings or with that of the double C=N bond. The structures were subjected to the unconstrained geometry optimization that was followed by the vibrational analysis. Results of the calculations with B3LYP functional were used for computing the Raman spectra of the complexes. Details of spectra calculations can be found elsewhere [42,49].

The calculations of the SA@M complexes were carried out for the enol, cis-keto, and trans-keto tautomers of the SA molecule. The stabilization energy ΔE of a complex was obtained as

(2) $$\Delta E=E_{C}-\left(E_{i}+E_{M}\right),$$

where $E_{C}$ is the energy of the complex, and $E_{i}$ and $E_{M}$ stand for the energies of the *i*th tautomer of the SA molecule and of the cation *M* in a free state, respectively. The energy $E_{C}$ was tested against the basis set superposition error (BSEE), which was found to be 0.5–0.7 kcal/mol at the B3LYP level. The error is smaller than the ΔE energies by at least an order of magnitude.

## 3. Results

### 3.1. DRUVv Spectra

**Figure 2:**
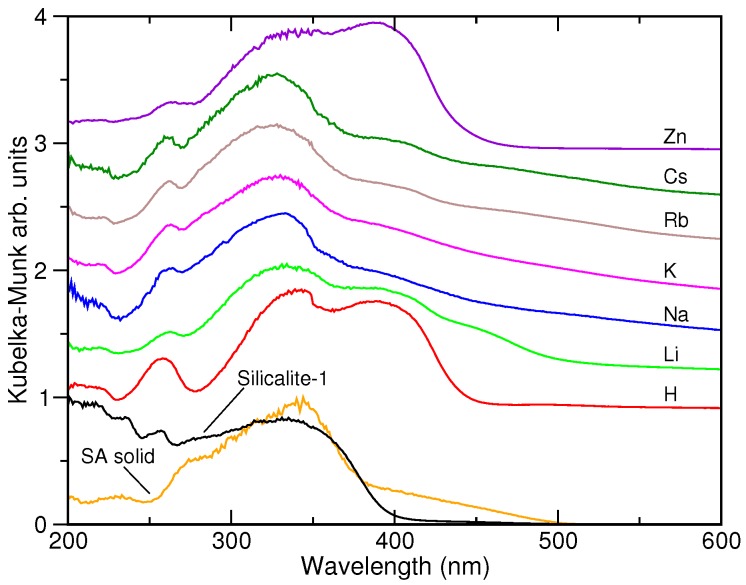
Diffuse reflectance UV-vis spectra of SA@M-ZSM-5 samples.

The DRUVv spectra of the SA@M-ZSM-5 systems are shown in Figure 2, where they are compared to the spectrum of SA in silicalite-1 and to that of the molecule in a solid state. The latter spectrum has a broad band between 250 and 380 nm with a maximum at ca. 350 nm and a shoulder at 270 nm. The absorption has a long tail extending to 500 nm. The spectrum of the SA@silicalite system is generally similar to the spectrum of the solid SA sample. Spectra of SA in the cation-exchanged ZSM-5 zeolites resemble each other and they are characterized by a wide band with the maximum at about 340 nm that shifts to 330 nm while going from H^+^ to Cs^+^ exchanged structures. A second band is present at 390 nm either as a well-defined feature, e.g., for H-ZSM-5 and Zn-ZSM-5, or as a shoulder. One could also infer a weak and wide band at about 450 nm in the spectra of some alkali-metal SA@M-ZSM-5 systems, e.g., M = Li^+^, and a long absorption tail extending up to 550–600 nm (see spectra of the SA@Rb,Cs-ZSM-5 systems in Figure 2). Finally, spectra of all SA@zeolite samples show the presence of a band at 260 nm.

### 3.2. Vibrational Spectroscopy

#### 3.2.1. Raman Spectra

**Figure 3:**
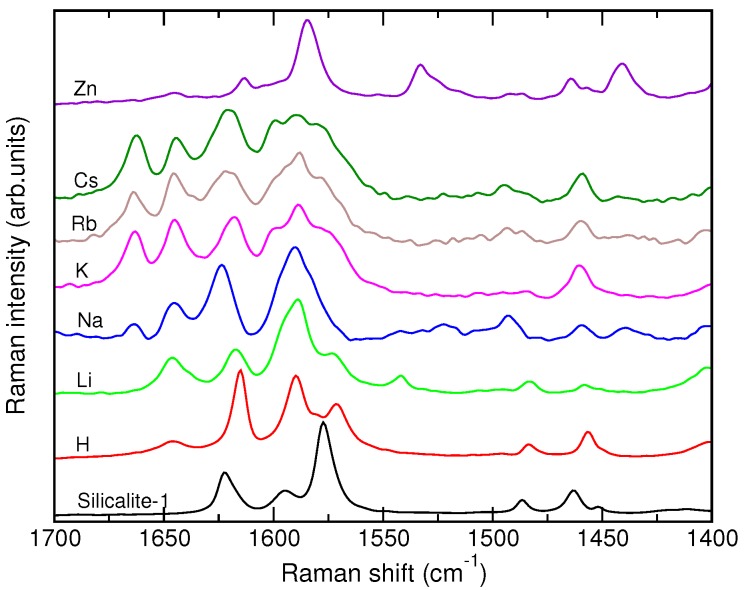
Experimental off-resonance Raman spectra of SA@M-ZSM-5 samples.

Figure 3 displays the off-resonance Raman spectra of SA@M-ZSM-5 samples and compares them with the Raman spectrum of SA in silicalite-1. The most informative part is the region from 1400 to 1700 cm${}^{-1}$, where the spectrum of SA@silicalite sample is characterized by peaks at 1622, 1594, 1577, 1486 and 1463 cm${}^{-1}$. Compared to the SA@silicalite spectrum, the spectra of SA in the M-ZSM-5 zeolites (M = H^+^, Li^+^, Na^+^, K^+^, Rb^+^, Cs^+^) have a number of new features. All the SA@M-ZSM-5 spectra contain a new peak at 1642 cm${}^{-1}$. Furthermore, the incorporation of the molecule into the Na^+^- to Cs^+^-exchanged materials is accompanied by the appearance of a peak at 1664 cm${}^{-1}$ that progressively grows in intensity from SA@Na-ZSM-5 to SA@Cs-ZSM-5 (Figure 3). Finally, the Raman peak observed at 1594 cm${}^{-1}$ in the spectrum of SA@silicalite changes its shape and reveals a complex structure in the spectra of all the SA@M-ZSM-5 samples with alkali-metal cations.

The spectral signature of SA in Zn-ZSM-5 markedly differs from that of the molecule occluded in the alkali-metal ZSM-5 zeolites (Figure 3). The spectrum of the SA@Zn-ZSM-5 sample is characterized by a strong peak at 1585 cm${}^{-1}$ that is accompanied by new peaks at 1533 and 1441 cm${}^{-1}$; the peak at 1642 cm${}^{-1}$ has a very low intensity in the spectrum of the system.

#### 3.2.2. DRIFT Spectra

**Figure 4:**
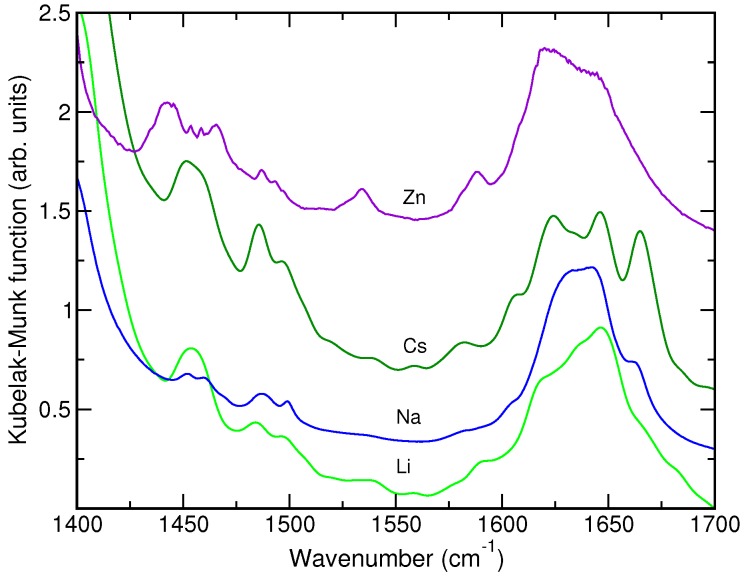
Experimental DRIFT spectra of the SA@silicalite and SA@M-ZSM-5 samples with M = Li^+^, Na^+^, Cs^+^, Zn^2+^.

Unfortunately, a strong absorption by the zeolite framework heavily limits the usability of the DRIFT spectra in the analysis of the SA@M-ZSM-5 systems. The region below 1400 cm${}^{-1}$ is completely hidden by the absorption of lattice modes and a broad band of variable intensity due to the overtone of T-O symmetric stretching modes is present at ca. 1630 cm${}^{-1}$. Figure 4 presents most representative DRIFT spectra of the SA@M-ZSM-5 samples (M = Li^+^, Na^+^, Cs^+^) that reveal the presence of bands at 1643 and 1663 cm${}^{-1}$. The latter band grows in relative intensity with increasing the size of the extra-framework cation. The vibrational signature of SA species stabilized in the presence of Zn^2+^ ion, which were observed in the Raman spectrum at 1533 and 1441 cm${}^{-1}$, are also clearly visible in the DRIFT spectrum of SA@Zn-ZSM-5 (Figure 4).

### 3.3. Quantum-Chemical Calculations

#### 3.3.1. Geometry and Stability of the SA@M Complexes

**Figure 5:**
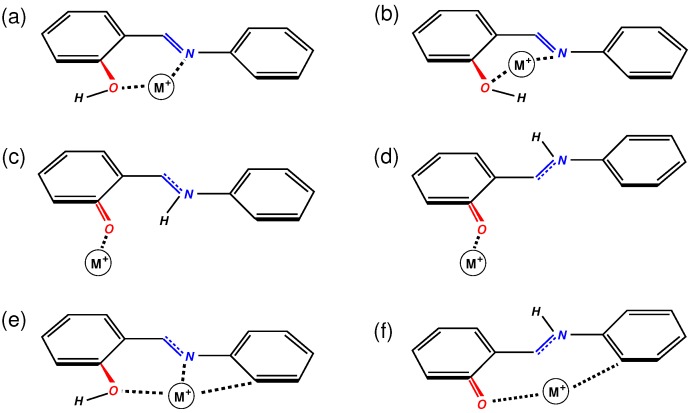
Optimized geometries of complex C: (**a**) enol SA tautomer with the Li^+^ and Na^+^ ions; (**b**) enol SA tautomer with K^+^ ion; (**c**) cis-keto SA tautomer with the ions; (**d**) trans-keto SA tautomer with the ions; (**e**) enol SA tautomer with Zn^2+^ ion; and (**f**) trans-keto SA tautomer with Zn^2+^ ion (ωB97X-D calculation).

Table 1 lists the stabilization energies ΔE (2) of the SA@M complexes computed at different theory levels. The geometry optimization of SA complexes A and B with the alkali-metal cations has led to final structures close to the initial configurations in Figure 1 for most of the SA conformers. In these complexes, the cation lies above the corresponding ring and interacts with the π electron system. Contrarily, the structural optimization of complexes C (Figure 1) has produced configurations with the cation moved away from its initial position and binded to the O and N atoms of the SA molecule. The structures of the most stable complexes C are depicted in Figure 5 and all the calculations have yielded the trans-keto SA tautomer complex C to be the most stable (Table 1 and Figure 5d). The complexes stability generally follows the tendency C > A > B and one can see that the stabilization energy diminishes with the increase of the cation size for each complex type. Note that the optimization of complexes A and B of the keto-SA forms with the Na^+^ and K^+^ ions results in the formation of complex C, in particular with the ωB97X-D functional. Coordinates of atoms in the structures shown in Figure 5 can be found in Appendix A.

The complexes of the SA molecule with the Zn^2+^ ion have stabilization energies largely superior (in absolute values) to those of the molecule with alkali-metal cations (Table 1). Complex C of the trans-keto SA tautomer (Figure 5d) was obtained to be the most stable at the B3LYP level, whereas the calculations at ωB97XD/6-311++G(d,p) yielded the largest stabilization energy for complex C of the enol SA form (Figure 5e) closely followed by complexes A and C (Figure 5f) of trans-keto SA (Table 1).

#### 3.3.2. Dipole Moment of SA Molecule

The dipole moments of SA conformers in a free state are equal to 2.35 D, 3.88 D, and 5.55 D for the enol, cis-keto, and trans-keto forms, respectively (B3LYP calculations). Assuming that a cation M can be approximated by a point charge $q_{M}$, the dipole moment of the SA molecule $\mu_{SA}$ in a SA@M complex can be estimated from the total dipole $\mu_{QC}$ obtained in quantum-chemical calculations as

(3) $$\mu_{SA}=\mu_{QC}-q_{M}r_{M},$$

where $r_{M}$ is the coordinate vector of the cation. Values of the molecular dipole in the SA@M complexes A to C with the Li^+^, Na^+^, and K^+^ ions computed for $q_{M}=+1\;\left|e^{-}\right|$ are gathered in Table 2.

Of course, the dipole values in Table 2 depend on the choice of charge $q_{M}$ that was arbitrarily set to the formal cation charge. Computation of charges in the complexes with the use of AIM topological analysis [50,51] resulted in a charge of the alkali-metal cations from +0.91 to +0.96 $|e^{-}|$, i.e., very close to the $q_{M}$ value used in Equation (3). Hence, the values reported in Table 2 can be considered as good estimates of the SA dipole moment in the SA@M complexes.

## 4. Discussion

### 4.1. DRUVv Spectra

Previous experimental study of SA in silicalite-1 has shown that the sorbed molecule has the enol conformation and that this Al-free solid behaves as an apolar solvent [42]. The 340 nm band in the spectrum of the SA@silicalite system is analogous to the absorption band observed at 350 nm for the molecule in solid state and in apolar and aprotic solvents. The band is characteristic of the π-$\pi^{*}$ transition of enol SA form [5,11,12]. The keto-enol tautomeric equilibrium of SA is known to be shifted towards a keto form in polar solvents [11,12,27] and such a behavior has been confirmed by quantum-chemical calculations using the polarizable continuum model [34]. Based on UV-visible spectra reported for anils in solutions or in confined state, the band at 390 nm in the spectra of SA@M-ZSM-5 samples can be ascribed to the π-$\pi^{*}$ transition of cis-keto SA tautomer or its zwitterionic form [3,12,13,24,35]. The presence of the band in the spectra of these systems suggests that the environment of the SA molecule in the zeolite void is polar enough to stabilize the keto form.

The broad and low intense features found above 400 nm are very similar to the residual absorption observed upon photo-excitation of SA in solutions and in confining media [27]. This contribution was assigned to a trans-keto SA tautomer created upon the relaxation of excited state of the cis-keto form [5,26,27,28]. However, to the best of our knowledge, this species has never been stabilized in solution and the attribution is solely based on results of the time-resolved spectroscopic measurements.

### 4.2. Vibrational Spectra

The Raman spectrum of SA sorbed in silicalite-1 is discussed in Ref. [42] in detail. According to the assignment proposed, the peaks at 1622 cm${}^{-1}$ and 1594 cm${}^{-1}$ arise from the C=N stretching vibration and from the 8a mode [52,53] localized on the benzene ring, respectively. The 1577 cm${}^{-1}$ peak is due to a mode involving the C=N, C−C, and C−O−H internal coordinates that accounts for the sensitivity of the mode to both the deuteration and ^15^N isotopic substitution [3]. Finally, the Raman peaks at 1486 and 1463 cm${}^{-1}$ come from the 18a mode [52,53] of the benzene ring and from a mode consisting of C−O and C−C bond-stretching vibrations of the phenol ring.

The spectra of SA sorbed in alkali-metal ZSM-5 zeolites strongly resembles spectra previously reported for the molecule in polar hydrogen-bonding solvents [3] and in NaY zeolites [23,24]. Analyzing Figure 3, one notices redistribution of the relative intensity of the C=N peak at 1622 cm${}^{-1}$ and of the new peak at 1642 cm${}^{-1}$. The peak at 1642 cm${}^{-1}$ has been assigned to a C=N^+^−H mode of the zwitterion of the cis-keto SA tautomer [3]. The blue frequency shift of the C=N mode upon the protonation of the imine group has been explained by a coupling between the C=N^+^ bond-stretching and the C=N^+^−H angle-bending vibrations [9,54]. Our quantum-chemical calculations [42] support this interpretation and show that the highly localized C=N mode in the enol form (96% of C=N bond-stretching force constant in the potential energy distribution) becomes delocalized over the vibrations of C=N, C−C, and C=N−H internal coordinates of the cis-keto conformer. Furthermore, according to the calculations, the 8a mode of the enol and cis-keto SA tautomers have vibrational frequencies of 1593 cm${}^{-1}$ and 1599 cm${}^{-1}$, respectively, that can account for the observed splitting of the 1594 cm${}^{-1}$ peak. Both the frequency values and their difference are in a very good agreement with the outcome of the Raman measurements. Therefore, the appearance of the peak at 1642 cm${}^{-1}$ and the complex structure of the peak at *ca.* 1590 cm${}^{-1}$ in the Raman spectra of the SA@M-ZSM-5 systems can be related to the formation of the cis-keto SA tautomer co-existing with the enol form in the pores of M-ZSM-5 zeolites.

The Raman spectra of SA@M-ZSM-5 samples with large alkali-metal cations (M = Na^+^, K^+^, Rb^+^, Cs^+^) show the appearance of peak at 1664 cm${}^{-1}$, in addition to the peak at 1642 cm${}^{-1}$ discussed above (Figure 3). The peak increases in intensity from Na- to Cs-containing structures and simultaneously, the 1590 cm${}^{-1}$ peak widens and reveals the presence of several (at least three) components. The new peak is likely to be a peak observed at 1656 cm${}^{-1}$ for different Schiff bases using the resonance Raman spectroscopy and seen as a shoulder at 1660 cm${}^{-1}$ in the infrared spectra [10]. A band in this spectral region has been found at 1651 cm${}^{-1}$ in the infrared spectra of SA in a mixed isopentane/methylcyclohexane solvent after flash photolysis [7] and at 1675 cm${}^{-1}$ in the IR spectrum of SA in CCl_4_ solution [55]. These spectral features have been assigned to the C=O stretching mode of the trans-keto tautomer of the SA molecule [7,10,55]. Very recently, the same attribution is suggested for a band at 1664 cm${}^{-1}$ observed in the IR spectra of SA derivatives in a polystyrene matrix upon irradiation [41]. Therefore, with a certain caution, the 1664 cm${}^{-1}$ peak in the spectra of M-ZSM-5 zeolites with large-size cations can be attributed to the presence of the trans-keto SA tautomer coexisting in the zeolite void with the other conformers.

The spectral signature of SA in Zn-ZSM-5 markedly differs from the spectra of the molecule occluded in the alkali-metal ZSM-5 zeolites, (Figure 3). The two peaks at 1533 and 1441 cm${}^{-1}$ feature in the Raman spectrum of this system, whereas other peaks have counterparts in the spectra of other SA@M-ZSM-5 systems. The difference can be related to a strong interaction of the Zn^2+^ ion with the molecule that results in the chelation process and affects the vibrational dynamics of the molecule to a larger extent compared to the case of monovalent alkali-metal cations. The SA@Zn-ZSM-5 spectrum is in line with Raman spectrum of 3-hydroxyflavone in a Zn-ZSM-5 zeolite that showed the appearance of two peaks at 1520 and 1465 cm${}^{-1}$ upon the complexation of the molecule with the Zn^2+^ ion [56].

Examination of the DRIFT spectra (Figure 4) yields the information that is generally in line with the results obtained by Raman spectroscopy.

### 4.3. General Discussion

The analysis of the set of the experimental spectroscopic data indicates that different forms of the salicylideneaniline molecule are stabilized in the M-ZSM-5 zeolite structures as a function of the size of charge-balancing cation M. The molecule is present as the enol conformer in a cation-free silicalite-1 structure, both the enol and cis-keto forms co-exist in the ZSM-5 voids with small-size cation, such as H^+^ and Li^+^. The increase of the cation size results in the appearance of spectral feature that can be ascribed to the trans-keto SA tautomer, which has previously been observed only as an illusive species in time-resolved spectroscopic experiments.

As it discussed above, the Al-free MFI structure provides a neutral environment for guest species and, thus, the SA molecule sorbed in silicalite-1 conducts itself as in a solid state [42]. One may then reasonably suppose that the molecule sorbed in the zeolite structure with a relatively low Al content will behave similarly. The role of the zeolite framework is then: (i) to accommodate the SA molecule in a specific part of the zeolite void; (ii) to impose constraints on the possible geometry of complexes of the molecule with extra-framework cations; and (iii) to favor, by local polar environment, the stabilization of a particular tautomer. In this respect, the siting sites of cations in the zeolite structure will play a crucial role.

The knowledge about the position of extra-framework cations in the ZSM-5 zeolites is scarce. Experimentally, the siting sites have been almost exclusively determined for Cs-exchanged ZSM-5 zeolite [57,58,59]. For the structure with the Si/Al = 15.6 ratio, i.e., similar to that in the present work, Olson et al. [57] found two of six cations in the intersections of straight and zigzag channels with the remaining Cs^+^ ions distributed over sites in the straight and zigzag channels. Similar results were obtained by Mentzen and co-workers [58] for Cs_6.6_-ZSM-5 structure, although with a slightly larger number of cations in the intersections. A computational study by Kucera and Nachtigall [60] shows that the small size Li^+^ ions prefer positions in channels of the ZSM-5 framework, whereas large K^+^ ions tend to occupy channel intersections. One may then argue that the molecule shares the channels with small-size alkali-metal cations, such as H^+^ and Li^+^, and with the Zn^2+^ ions whose size is similar to that of the lithium cation. On the other hand, as large cations obstruct the channels and do not leave enough room for the molecule and therefore, SA forms complexes with large-size cations situated in channel intersections.

Considering possible complexes of cis-keto SA with H^+^ and Li^+^ ions, the complexes C can be ruled out because such a complex (Figure 5) is too large to fit the space available in the MFI channels. Hence, we suggest that these are complexes A and B (Figure 1) of cis-keto tautomer that are formed and co-exist in the zeolite pores with the enol SA form, which is present in cation-free regions of the void. The cis-keto tautomer was proposed to exist in the zeolite void as a zwitterionic structure [3,23,24,35]. The dipole of isolated SA conformers are far from the glycine zwitterion dipole of 12 Debye [61], which is often considered as a reference value of zwitterion dipole. The analysis of Table 2 shows that the molecular dipole of the enol form in SA@M complexes and the dipole values of all SA tautomers in complex B remain notably below the reference value. On the other hand, the keto forms of the molecule in complexes A and C have indeed values of the dipole close to that of a zwitterion. Then, under the assumption on the stabilization of complex A in the ZSM-5 structures with small-size extra-framework cations, one may suppose that the zwitterionic structure of the cis-keto SA tautomer can exist in the SA@H-ZSM-5 and SA@Li-ZSM-5 systems.

**Figure 6:**
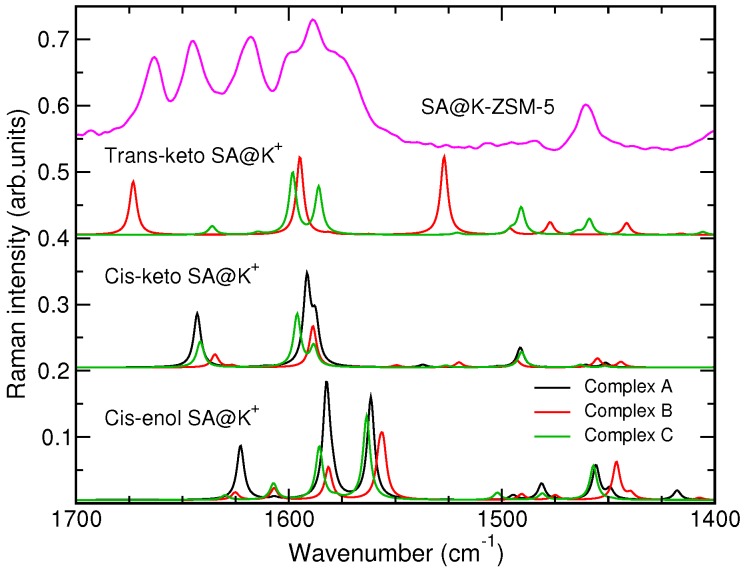
Calculated Raman spectra of SA@K^+^ complexes and the experimental spectrum of SA@K-ZSM-5 sample in the zone 1400–1700 cm${}^{-1}$. No scaling factor was applied to the peak intensity in the computed spectra.

According to the discussion above, the presence of large-size cations in the void of the MFI framework may stabilize the trans-keto tautomer of the SA molecule whose spectral signature is the peak at 1664 cm${}^{-1}$ in the Raman spectrum. Figure 6 presents calculated Raman spectra of complexes of the SA molecule with K^+^ ion. The inspection of the spectra shows that the only complex that has a Raman peak in the zone above 1660 cm${}^{-1}$ is complex B of the trans-keto SA tautomer with the cation. Therefore, results of the calculations support the conclusion drawn from the analysis of the experimental data about the stabilization of this species in ZSM-5 zeolites with large-size extra-framework cations. According to Ref. [60], increasing cation’s size leads to preferential population of channel intersections of the MFI framework by cations. It is, therefore, reasonable to relate the stabilization of the trans-keto SA form to the presence of the cations in the channel intersections.

Of course, the results of the calculations should be taken into account with a certain degree of caution since they were performed for free complexes and, therefore, do not take into account the effect of confinement by the zeolite lattice. It is, however, worth noting that, regardless the cation, the only system with a vibrational mode above 1660 cm${}^{-1}$ is the trans-keto tautomer in the SA@M complexes. The analysis of the potential energy distribution in the corresponding mode shows that the dominant contribution, ca. 85 %, comes from the C=O bond-stretching coordinate that is in a good agreement with the assignment suggested for this mode in Refs. [7,10,55]

## 5. Conclusions

Salicylideneaniline sorbed in alkali-metal exchanged ZSM-5 zeolites was studied by means of electronic and vibrational spectroscopies. Results of the investigation show that, in contrast to SA sorbed in silicalite-1 (an Al-free counterpart of ZSM-5 zeolites), the state of the molecule sorbed in the aluminosilicate structures reveals a complex behavior depending on the nature of extra-framework cation. Quantum-chemical calculations of SA–alkali-metal cation complexes helped to better understand the experimental findings.

In line with literature data [23,24,35], the presence of cations in the zeolite pore system produces an environment for the SA molecule, similar to that in polar solvents. The results obtained for the SA@M-ZSM-5 zeolites with small size cations, such as H^+^ and Li^+^, point to the stabilization of a cis-keto SA tautomer that co-exists with a enol one in the zeolite void. The calculations indicate that the cis-keto SA tautomer in the complexes with small-size cations has a large dipole value that is close that expected for a zwitterionic form. The stabilization energies of the complexes and the dipole of the molecule were found to decrease with the increase of the cation size and depend on the complex’s geometry. Increase of the cation size leads to the appearance of a new peak at 1664 cm${}^{-1}$ in the Raman spectra that was ascribed to a trans-keto tautomer of salicylideneaniline that is stabilized in the zeolite pores along with the enol and cis-keto conformers. The stabilization of the trans-keto form of SA, which has previously been observed only on time-resolved experiments, is presumably due to a specific geometry of the cation-SA complex in a tight confinement by relatively rigid zeolite framework with the extra-framework cations present in the intersections of the straight and zigzag channels. Data concerning the sorption of salicylideneaniline in the Zn-ZSM-5 structure can be interpreted by the chelation between the divalent extra-framework cations and the enol form of the molecule.

The results of the study imply that the tautomeric equilibrium of molecules belonging to the Schiff base family can be tuned via a confinement in zeolites by a judicious choice of both the topology of zeolite pore system and the nature of extra-framework cations.

## Figures and Tables

**Table 1:** Stabilization energies ΔE (in kcal/mol) of SA@M complexes A to C (Figure 1) with the Li^+^, Na^+^, K^+^ and Zn^2+^ ions ^*a*^.

	Enol			Cis-keto			Trans-keto		
	A	B	C	A	B	C	A	B	C
Li^+^	−40.9	−40.3	−59.0 (a) ${}^{b}$	−45.0	−38.0	−62.8 (c)	−43.7	−36.4	−69.3 (d)
	−39.7	−38.7	−56.6 (a)	−44.2	−36.9	−60.3 (c)	−42.8	−35.7	−65.8 (d)
Na^+^	−25.5	−24.5	−36.0 (a)	−29.2	−22.9	−44.2 (c)	−28.6	−21.4	−49.6 (d)
	−26.5	−25.0	−37.4 (a)	−30.5	−24.0	−43.2 (c)	− ${}^{c}$	−22.9	−47.7 (d)
K^+^	−17.1	−15.8	−20.7 (b)	−20.8	−15.2	−32.6 (c)	− ${}^{c}$	−13.8	−38.0 (d)
	−19.9	−18.2	−23.4 (b)	−23.5	− ${}^{c}$	−33.4 (c)	− ${}^{c}$	− ${}^{c}$	−38.0 (d)
Zn^2+^	−207.7	−211.7	−236.0 (e)	−228.5	− ${}^{d}$	−233.3 (c)	−232.3	− ${}^{d}$	−242.9 (d)
	−184.7	−193.6	−228.7 (e)	−219.8	−191.7	− ^*e*^	−226.2	− ${}^{c}$	−227.5 (f)

^*a*^ The lower and upper entry for each complex corresponds to the ΔE value obtained at the B3LYP and ωB97X-D levels, respectively. ^*b*^ The letter in parentheses refers to the corresponding panel in Figure 5. ^*c*^ Transforms to complex C upon geometry optimization. ^*d*^ No stable configuration for this complex was found. ^*e*^ Transforms to complex B upon geometry optimization.

**Table 2:** Dipole moment (in Debye) of the SA molecule in complexes A to C (Figure 1) with the Li^+^, Na^+^ and K^+^ ions computed at B3LYP level.

	Li^+^			Na^+^			K^+^		
	A	B	C	A	B	C	A	B	C
Enol SA	7.99	6.50	6.58	6.93	5.49	6.44	6.22	4.82	6.04
Cis-keto SA	10.82	7.11	10.69	9.69	6.14	9.77	8.95	5.66	9.45
Trans-keto SA	12.09	7.32	13.48	11.27	6.81	12.18	− ${}^{a}$	6.70	11.83

^*a*^ Transforms to complex C upon geometry optimization.

## Abbreviations

The following abbreviations are used in this manuscript:

MAS	Magic-angle spinning
NMR	Nuclear magnetic resonance
IR	Infrared
DRUVv	Diffuse reflectance ultraviolet-visible
DRIFT	Diffuse reflectance infrared Fourier transform

## Appendix A. Coordinates of Atoms (x y z, in Å) in SA@M Complexes

### Appendix A.1. Enol SA@Li (Figure 5a), B3LYP Optimized Geometry

Li	−0.108117	2.207140	0.407782
C	3.809128	0.795021	0.168986
C	2.422832	0.788320	0.165361
C	1.693700	−0.388898	−0.095454
C	2.441717	−1.557299	−0.349199
C	3.827594	−1.562122	−0.349000
C	4.514158	−0.378474	−0.088096
H	4.347077	1.716309	0.372584
O	1.745173	1.984038	0.432620
H	2.398490	2.669674	0.624534
H	1.903220	−2.476370	−0.551158
H	4.369076	−2.477677	−0.549124
H	5.597102	−0.360518	−0.082364
C	0.242817	−0.527305	−0.130940
H	−0.104567	−1.546850	−0.313774
C	−3.905992	−1.250689	0.648640
C	−2.534489	−1.013055	0.666218
C	−2.014242	0.104830	0.002777
C	−2.880585	0.989700	−0.651060
C	−4.249211	0.739706	−0.672097
C	−4.764811	−0.380882	−0.022028
H	−4.305387	−2.111268	1.171992
H	−1.878229	−1.671388	1.223788
H	−2.478241	1.845659	−1.185427
H	−4.911374	1.417135	−1.197782
H	−5.831249	−0.570344	−0.030469
N	−0.622663	0.416970	0.009749

### Appendix A.2. Enol SA@K (Figure 5b), B3LYP Optimized Geometry

K	−0.282699	2.458482	0.982719
C	3.716504	0.619429	−0.456434
C	2.341772	0.417328	−0.465229
C	1.790474	−0.830179	−0.080434
C	2.672628	−1.866006	0.286044
C	4.042858	−1.665749	0.297229
C	4.559478	−0.419135	−0.070740
H	4.110744	1.578432	−0.769674
O	1.521339	1.448180	−0.849206
H	0.645896	1.031831	−1.034418
H	2.260856	−2.829781	0.566039
H	4.709487	−2.469200	0.583326
H	5.631179	−0.258465	−0.069823
C	0.359193	−1.064505	−0.054694
H	0.038259	−2.081674	0.187994
C	−3.844318	−1.394036	0.825741
C	−2.464134	−1.219425	0.766973
C	−1.904253	−0.390323	−0.216908
C	−2.748012	0.277770	−1.116179
C	−4.125835	0.089329	−1.055929
C	−4.678004	−0.746214	−0.085365
H	−4.269543	−2.034211	1.589809
H	−1.828418	−1.711581	1.494652
H	−2.311920	0.903069	−1.888313
H	−4.767678	0.589910	−1.771257
H	−5.750737	−0.888194	−0.036063
N	−0.511096	−0.139492	−0.310676

### Appendix A.3. Cis-keto SA@K Complex C (Figure 5c), B3LYP Optimized Geometry

K	−2.081024	3.400573	−0.276893
C	−3.439256	−0.242264	0.093048
C	−2.022722	−0.112680	0.067373
C	−1.265049	−1.348407	0.062953
C	−1.938768	−2.602958	0.079499
C	−3.307301	−2.675342	0.100187
C	−4.053556	−1.475404	0.108372
H	−4.039160	0.662105	0.109077
O	−1.439048	1.044169	0.047806
H	−1.343939	−3.510729	0.076542
H	−3.812898	−3.631932	0.112566
H	−5.136847	−1.527036	0.129523
C	0.142785	−1.348953	0.053756
H	0.669590	−2.298369	0.049661
C	4.452031	−1.051052	−0.605435
C	3.064838	−1.163196	−0.603053
C	2.297818	−0.181661	0.029621
C	2.916127	0.917633	0.631836
C	4.303013	1.024243	0.614912
C	5.074888	0.038616	0.001559
H	5.046179	−1.810862	−1.098744
H	2.590785	−1.991880	−1.115112
H	2.314804	1.668094	1.133410
H	4.780835	1.872757	1.089740
H	6.154734	0.121917	−0.009062
N	0.880496	−0.256221	0.068008
H	0.315196	0.603796	0.117098

### Appendix A.4. Cis-keto SA@Na Complex C (Figure 5d), B3LYP Optimized Geometry

Na	2.835588	3.549777	−0.351181
C	2.985303	−2.495177	0.181006
C	1.694005	−2.052405	0.144525
C	1.388054	−0.660272	0.045504
C	2.465943	0.318199	−0.017586
C	3.798707	−0.202707	0.032292
C	4.043409	−1.548203	0.122937
H	3.207451	−3.551121	0.263697
H	0.891426	−2.780363	0.212412
O	2.238977	1.572532	−0.118880
H	4.619356	0.504985	−0.007850
H	5.068230	−1.902157	0.155441
C	0.080076	−0.164251	0.010984
H	−0.041552	0.912277	0.002530
C	−3.991557	1.283493	0.653997
C	−2.676229	0.827856	0.652862
C	−2.369888	−0.374184	0.011629
C	−3.375401	−1.130482	−0.595241
C	−4.687862	−0.670263	−0.577018
C	−4.998777	0.540277	0.040365
H	−4.232531	2.212219	1.157246
H	−1.910037	1.386369	1.176658
H	−3.131584	−2.064695	−1.090153
H	−5.465891	−1.257093	−1.049943
H	−6.021621	0.896141	0.054346
N	−1.037578	−0.876169	−0.026322
H	−0.958177	−1.882471	−0.113652

### Appendix A.5. Enol SA@Zn Complex C (Figure 5e), B3LYP Optimized Geometry

Zn	−0.560748	−1.441577	−0.032947
C	3.687952	−0.855141	−0.177048
C	2.356325	−0.505883	−0.149293
C	1.938127	0.837591	0.053456
C	2.961867	1.806587	0.188992
C	4.303622	1.466101	0.159780
C	4.666666	0.133275	−0.019384
H	3.989792	−1.886945	−0.331459
O	1.409546	−1.561649	−0.387232
H	1.886199	−2.355047	−0.680277
H	2.673443	2.841526	0.334751
H	5.062366	2.229627	0.274389
H	5.711422	−0.152646	−0.048225
C	0.582404	1.308370	0.152545
H	0.423685	2.387265	0.152631
C	−3.893362	1.654508	−0.498349
C	−2.510205	1.780399	−0.298661
C	−1.820619	0.701532	0.225083
C	−2.528575	−0.496376	0.605494
C	−3.916088	−0.604856	0.359351
C	−4.586303	0.471086	−0.203848
H	−4.433718	2.488754	−0.932288
H	−1.997700	2.679736	−0.618773
H	−2.156742	−1.042073	1.488345
H	−4.455540	−1.491290	0.672075
H	−5.651711	0.411548	−0.389404
N	−0.422327	0.492413	0.253685

### Appendix A.6. Enol SA@Zn Complex C (Figure 5f), ωB97X-D Optimized Geometry

Zn	−0.460085	−1.428863	−0.153088
C	3.959947	1.514066	−0.354040
C	2.658946	1.786650	−0.094714
C	1.786369	0.699063	0.254940
C	2.254009	−0.689940	0.082063
C	3.624583	−0.918987	−0.105696
C	4.438266	0.162465	−0.333566
H	4.660961	2.315195	−0.551440
H	2.305630	2.809547	−0.033532
O	1.396566	−1.661441	−0.029988
H	3.989741	−1.935885	−0.175039
H	5.487914	−0.012082	−0.546127
C	0.531549	0.769233	0.792674
H	0.265120	−0.081297	1.410174
C	−3.337839	−0.851725	0.245075
C	−2.037762	−0.341756	0.632366
C	−1.757982	1.070680	0.355401
C	−2.713537	1.838004	−0.289418
C	−3.920654	1.267627	−0.678933
C	−4.233382	−0.082267	−0.439825
H	−3.599204	−1.860607	0.547799
H	−1.759686	−0.673545	1.644008
H	−2.525463	2.885937	−0.496372
H	−4.649358	1.897271	−1.179406
H	−5.193291	−0.480539	−0.742602
N	−0.505707	1.631859	0.623605
H	−0.327485	2.531726	0.191905
